# Dataset on characterization of hemin-azide derivative and DNA oligonucleotide-hemin conjugate

**DOI:** 10.1016/j.dib.2017.05.020

**Published:** 2017-05-13

**Authors:** J. Kosman, A. Stanislawska, A. Gluszynska, B. Juskowiak

**Affiliations:** Laboratory of Bioanalytical Chemistry, Faculty of Chemistry, Adam Mickiewicz University, Umultowska 89b, 61–614 Poznan, Poland

## Abstract

In this article newly synthesized azide derivative of hemin and DNA-hemin conjugate are characterized. Hemin-azide was purified using HPLC and characterized using elemental analysis, IR and NMR. The DNA-hemin conjugate was obtained via click chemistry [1] and click reaction was carried out using traditional Cu-catalyzed and Cu-free approaches. The final product was successfully obtained using Cu-free cycloaddition. The identity of product was confirmed using Maldi TOF spectrometry. Obtained hemin-DNA conjugate exhibited peroxidase-like activity.

**Specifications Table**

**Table:** 

Subject area	*Chemistry*
More specific subject area	*Modification of bioorganic molecules*
Type of data	*Figures, Tables*
How data was acquired	*HPLC,*^*13*^*C NMR, IR, MALDI TOF, microplate reader*
Data format	*Raw, analyzed*
Experimental factors	*Characterization of product of hemin modification with azide group. Characterization of DNA oligonucleotide-hemin conjugate obtained by click reaction.*
Experimental features	*Experiments were performed using PS2.M DNA sequence: 5’-GTGGGTAGGGCGGGTTGG-3’.*
Data source location	*Adam Mickiewicz University, Poznan, Poland*
Data accessibility	*The data are provided with this article.*

**Value of the data**

• Presented in this paper data set focuses on characterization of newly synthesized hemin-azide derivative and DNA-hemin conjugates obtained by click reaction. This new method of DNA-hemin synthesis gives high yield and do not require sophisticated lab equipment [1].
• Presented data can be useful for researchers who would like to synthesize DNA oligonucleotide-hemin conjugates using described by us procedure.
• Obtained new DNAzyme, based on hemin-DNA oligonucleotide synthesized by click reaction, is a new system for bioanalytical applications.

## 1 Data

**Fig. 1:**
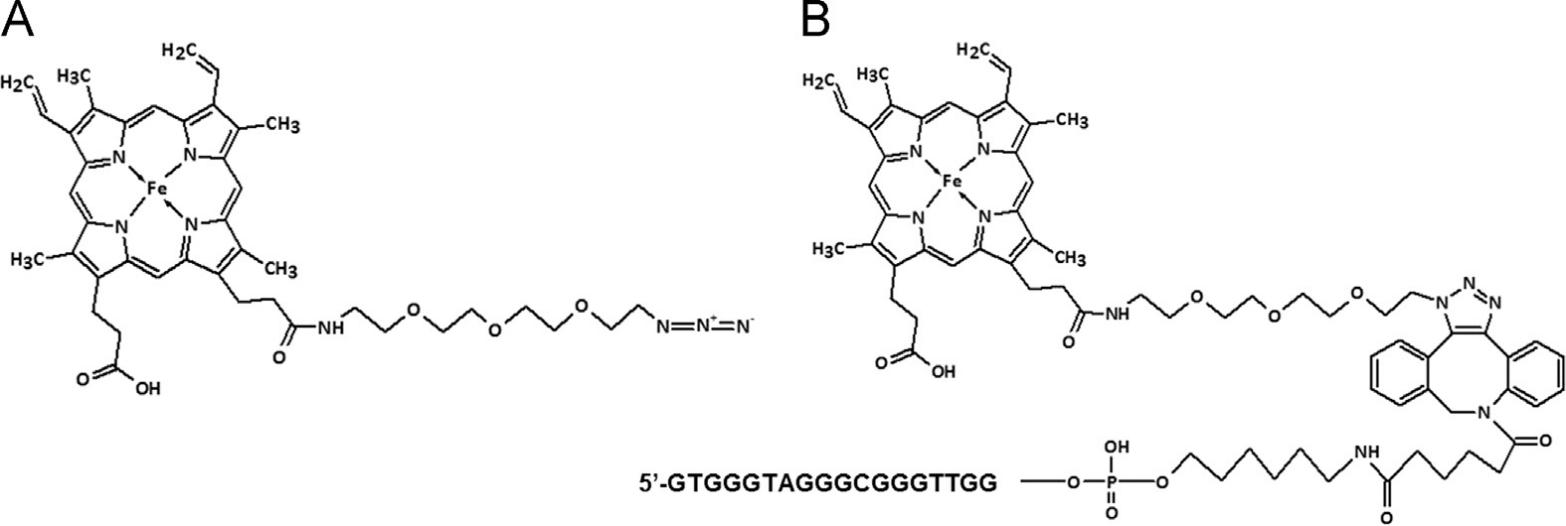
Scheme of the structure of hemin-azide (A) and DNA oligonucleotide-hemin conjugate (B).

**Fig. 2:**
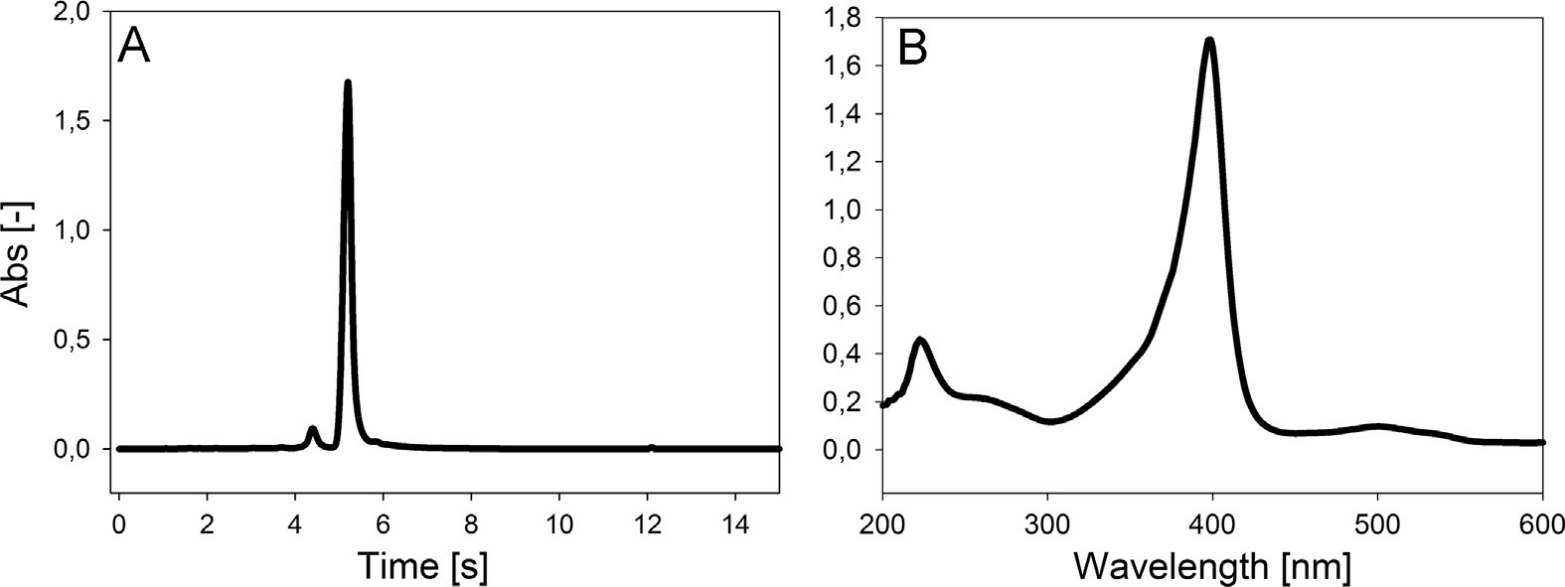
HPLC chromatogram (A) and UV-Vis spectrum (B) of hemin-azide derivative.

**Fig. 3:**
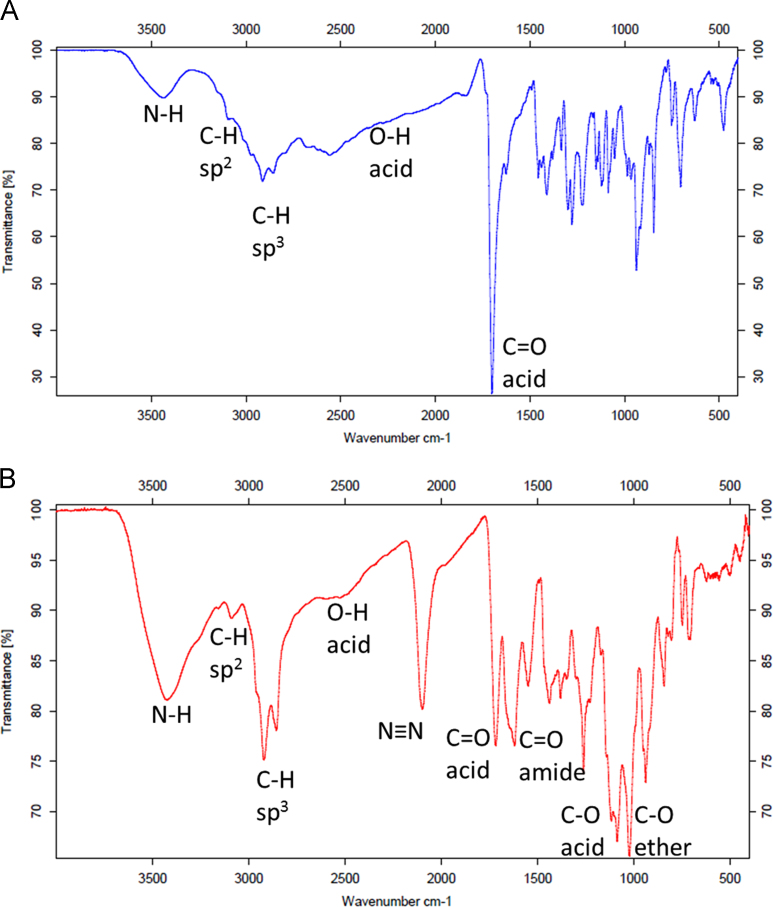
IR spectra of hemin (A) and hemin-azide (B).

**Fig. 4:**
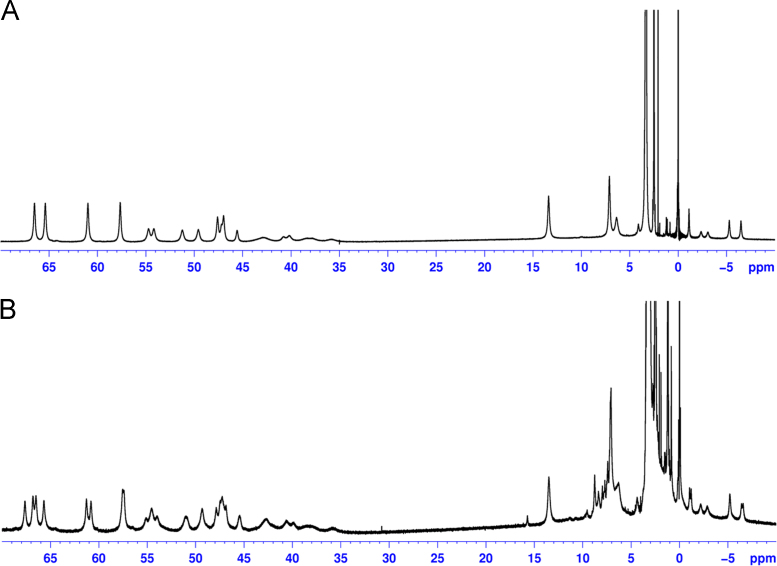
^1^H NMR spectra of hemin (A) and hemin-azide (B).

**Fig. 5:**
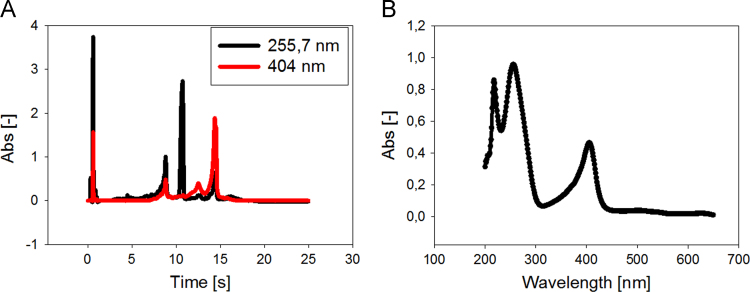
HPLC chromatogram of CuAAC synthesis mixture (A). R_t_ = 8.8 min corresponds to PS2.M-hem, R_t_ = 10.7 min to unreacted DNA and R_t_ = 14.4 to unreacted hemin-azide. UV-Vis spectrum of CuAAC reaction product with R_t_ = 8.8 min (B).

**Fig. 6:**
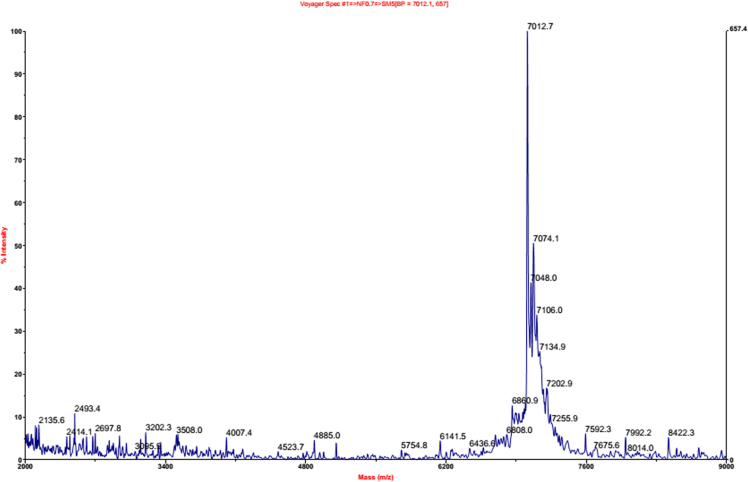
Mass spectrum of PS2.M-hem conjugate (**4**). Calculated mass is 7018 m/z, found mass is 7012.7.

**Fig. 7:**
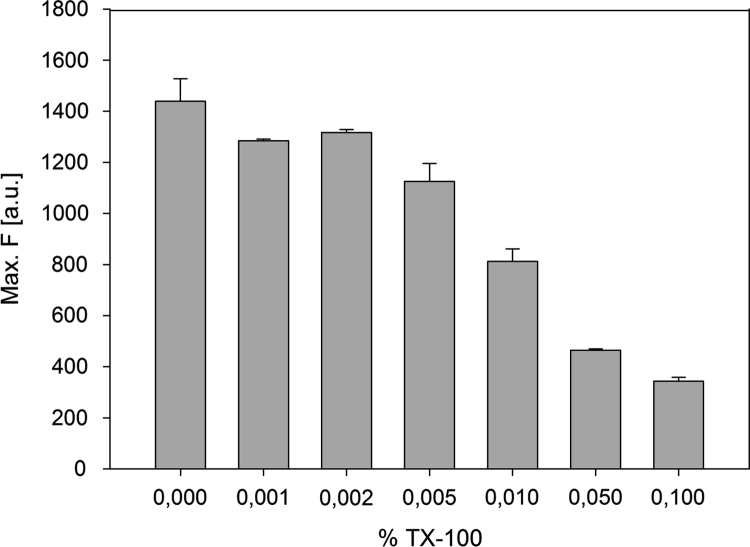
Influence of surfactant concentration on peroxidase activity of PS2.M-hem DNAzyme. Conditions: 10 mM Tris-HCl, 100 mM KCl, 0 – 0.1% Triton X-100, 50 nM DNA, 50 nM hemin (if present), 10 μM MNBDH, 1 mM H_2_O_2_.

Data presented here describes the characterization of hemin-azide and DNA oligonucleotide-hemin conjugate (Fig. 1). Associative complex between hemin and DNA oligonucleotide (that forms G-quadruplex structure) is known of its peroxidase-like activity [2]. This DNAzyme found great application in bioanalysis in detection of DNA sequences, proteins and metal ions [3]. First step involved synthesis of hemin-azide derivative. Hemin modification was performed using commercially available oxyethylene connector with amine group on one side and azide group on the other end. Synthesis was performed by amine coupling reaction. Successful conjugation of hemin-azide to DNA oligonucleotide was performed using Cu-free click chemistry [1]. The presented results are the first data on characterization of conjugation of hemin to DNA oligonucleotide using click chemistry. Synthesized hemin-azide substrate for click reaction was purified using HPLC (Fig. 2). Hemin-azide was then characterized using elemental analysis (Table 1), IR (Fig. 3) and NMR (Fig. 4). The synthesis of hemin-DNA oligonucleotide conjugate was performed first using CuACC reaction (Copper(I)-catalyzed alkyne-azide cycloaddition). Many variants of the conditions for click reaction have been used (Table 2). The hemin-DNA conjugate was purified using HPLC (Fig. 5). However the final product of the reaction was not soluble in water or organic solvents. The hemin-DNA oligonucleotide conjugate was successfully synthesized using Cu-free SPAAC reaction (Strain-promoted alkyne-azide cycloaddition). Maldi TOF spectrometry was used to confirm the identity of obtained product (Fig. 6). Synthesized hemin-DNA conjugate exhibited peroxidase activity which did not require the presence of surfactants routinely used for the traditional hemin/DNA associative system (Fig. 7).

**Table 1:** Elemental analysis and mass spectrometry of hemin-azide derivative.

Elemental analysis			
	%C	%N	%H
Theoretical	59.21	13.16	5.64
Experimental	57.83	11.53	6.31
Mass spectrometry			
Theoretical	816.3		
Experimental	816.4

**Table 2:** Composition of reaction solution for CuACC approach.

	Cu derivative	Ligand/additional component
1	CuBr (2.5 mM)	TBTA (Tris[(1-benzyl-1*H* 1,2,3-triazol-4-yl)methyl]amine) (5 mM)
2	CuI (7.8 mM)	TBTA (15.6 mM), DiPEA (*N*,*N*-Diisopropylethylamine) (78 μM)
3	CuSO_4_ (1.2 mM)	TBTA (1.2 mM), sodium ascorbate (12 mM)
4	[Cu(ACN)_4_]PF_6_ (7.8 mM)	TBTA (15.6 mM), DiPEA (78 μM)

## 2 Experimental Design, Materials and Methods

The materials and methods used in this paper are described in [1].

## Figures and Tables

**Fig. S1:**
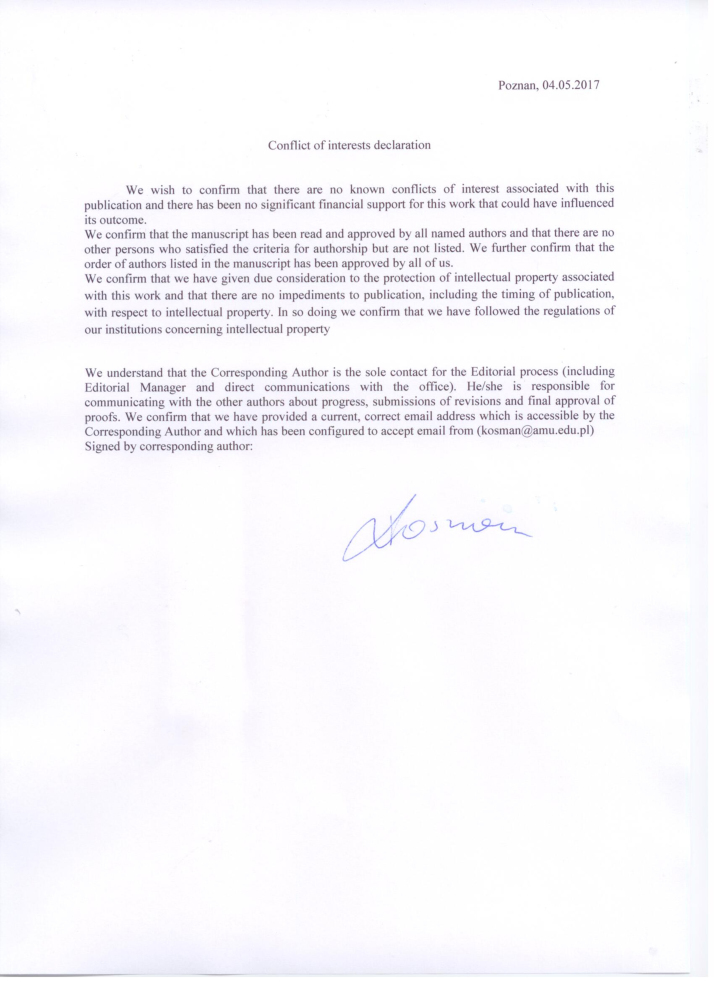
Supplementary material
